# A Live Attenuated H9N2 Avian Influenza Vaccine Prevents the Viral Reassortment by Exchanging the HA and NS1 Packaging Signals

**DOI:** 10.3389/fmicb.2020.613437

**Published:** 2021-02-04

**Authors:** Sujuan Chen, Keji Quan, Hui Wang, Shi Li, Jing Xue, Tao Qin, Dianfeng Chu, Gencheng Fan, Yuanzhao Du, Daxin Peng

**Affiliations:** ^1^College of Veterinary Medicine, Yangzhou University, Yangzhou, China; ^2^Jiangsu Co-Innovation Center for the Prevention and Control of Important Animal Infectious Disease and Zoonoses, Yangzhou University, Yangzhou, China; ^3^Joint International Research Laboratory of Agriculture and Agri-Product Safety, The Ministry of Education of China, Yangzhou, China; ^4^Jiangsu Research Centre of Engineering and Technology for Prevention and Control of Poultry Disease, Yangzhou, China; ^5^State Key Laboratory of Genetically Engineered Veterinary Vaccines, Qingdao Yibang Biological Engineering Co., Ltd., Qingdao, China

**Keywords:** avian influenza virus, H9N2 subtype, live-attenuated vaccine, HA, NS1, packaging signals

## Abstract

The H9N2 avian influenza virus is not only an important zoonotic pathogen, it can also easily recombine with other subtypes to generate novel reassortments, such as the H7N9 virus. Although H9N2 live attenuated vaccines can provide good multiple immunities, including humoral, cellular, and mucosal immunity, the risk of reassortment between the vaccine strain and wild-type virus is still a concern. Here, we successfully rescued an H9N2 live attenuated strain [rTX-NS1-128 (mut)] that can interdict reassortment, which was developed by exchanging the mutual packaging signals of HA and truncated NS1 genes and confirmed by RT-PCR and sequencing. The dynamic growth results showed that rTX-NS1-128 (mut) replication ability in chick embryos was not significantly affected by our construction strategy compared to the parent virus rTX strain. Moreover, rTX-NS1-128 (mut) had good genetic stability after 15 generations and possessed low pathogenicity and no contact transmission characteristics in chickens. Furthermore, chickens were intranasally immunized by rTX-NS1-128 (mut) with a single dose, and the results showed that the hemagglutination inhibition (HI) titers peaked at 3 weeks after vaccination and lasted at least until 11 weeks. The cellular immunity (IL-6 and IL-12) and mucosal immunity (IgA and IgG) in the nasal and trachea samples were significantly increased compared to inactivated rTX. Recombinant virus provided a good cross-protection against homologous TX strain (100%) and heterologous F98 strain (80%) challenge. Collectively, these data indicated that rTX-NS1-128(mut) lost the ability for independent reassortment of HA and NS1-128 and will be expected to be used as a potential live attenuated vaccine against H9N2 subtype avian influenza.

## Introduction

In China, H9N2 subtype low pathogenicity avian influenza virus (LPAIV) outbreak was first reported in Guangdong province in 1992 (Gu et al., 2017), which mainly causes mild respiratory symptoms, egg-drop symptoms, and immunosuppression (Li et al., 2005; Chen et al., 2013; Sun and Liu, 2015). Although the H9N2 virus usually shows low pathogenicity to birds, it can co-infect with other pathogens and aggravate symptoms and cause huge economic losses. Moreover, the first case of human infection with the H9N2 virus was reported in China in 1998, implying that it also can cross species barriers to infect humans (Gou et al., 2000; Saito et al., 2001; Perez et al., 2003). Additionally, the H9N2 virus can provide internal genes to new emerging reassortant viruses, such as the H7N9 virus outbreak in China in 2013 (Wu et al., 2013). Therefore, how to effectively prevent and control H9N2 LPAIV in poultry is crucial for both the poultry industry and public health security.

Currently, vaccination and biosecurity are used to control H9N2 LPAI in China. Since 1998, the inactivated vaccines have been licensed and widely used in chickens (Li et al., 2005). The inactivated vaccines only induce humoral immunity, which is not sufficient to completely control H9N2 LPAI. As the continued evolution of H9N2 viruses, differences of virulence and antigenicity resulted in vaccination failure despite a high level of antibody produced in immunized chickens (Zhang et al., 2008; Sun et al., 2012; Liu et al., 2016; Gu et al., 2017). Therefore, it is necessary to develop other vaccine types that can induce both humoral and cellular immunity at mucosal sites.

Previous studies have shown that LPAIV live attenuated vaccines have been demonstrated to generate broad long-lasting immunity and to provide cross-protection against different influenza viruses and can be administered conveniently (Nfon et al., 2012; Astill et al., 2018). Importantly, they can strongly elicit a humoral immune response and enhance the cellular and mucosal immunity even if antigens are at low doses (Suguitan et al., 2006; Joseph et al., 2008; Lee et al., 2008). Live attenuated vaccines for children used in Europe and the United States were considered better than inactivated vaccines (Falkenhorst et al., 2013; Grohskopf et al., 2014). Some attenuated cold-adapted live H9N2 subtype AIV vaccine strains have better safety and efficacy profiles (Chen et al., 2003; Lee et al., 2008; Wei et al., 2016a). Attenuated influenza viruses have been developed based on the deletion of truncation of the NS1 gene that interferes with interferon (Kochs et al., 2007). Previously, we developed a live attenuated H9N2 vaccine by truncating the NS1 gene (named rTX-NS1-128), which can protect chickens against homologous and heterologous H9N2 AIVs challenge (Chen et al., 2017). Therefore, the live attenuated vaccine has more advantages compared with the inactivated vaccine. However, H9N2 AIV provides a partial and even the whole set of internal genes to emerging human H5N1, H7N9, H10N8, and H5N6 reassortments recently (Guan et al., 2000; Gu et al., 2014; Huang et al., 2015; RahimiRad et al., 2016; Shen et al., 2016). Additionally, some experiments *in vivo* and *in vitro* confirmed that influenza viruses had a high recombination frequency (Marshall et al., 2013; Urbaniak et al., 2017; Zhou et al., 2017; Li et al., 2018). There is indeed concern about the risks associated with reassortment (or segment exchange) events between vaccine strains and circulating wild-type viruses. Therefore, it is necessary to consider how to prevent virus recombination in using live attenuated influenza vaccines in poultry.

The final step of the influenza virus forming a progeny virus requires packaging the different vRNA fragments into virions, which relies on the terminal packaging signal sequence of the vRNA. Therefore, the packaging signals of different gene segments of the influenza virus are swapped, which may be an attractive strategy to avoid reassortment between vaccine strains and wild-type viruses. Using green fluorescent protein (GFP), the packaging signal sequence of different gene fragments of AIV has been revealed, including 5′ and 3′ non-coding regions (NCRs) and part of the open reading frame (ORF) of 5′ and 3′ in the different genes (Goto et al., 2013). In the virus-assembly process, each packaging signal must ensure that the eight vRNA fragments are accurately packaged into progeny virions. The recombinant virus was rescued by exchanging HA and NS packaging signals, which cannot recombine freely with the NS and HA fragments of wild-type viruses (Gao and Palese, 2009). Therefore, the vRNA reassembling by replacing the packaging signal may be a feasible method to prevent the recombination of specific vRNA fragments. This strategy can be applied to develop live attenuated vaccines that have gene segments that are resistant to recombination.

In our previous study, H9N2 live attenuated vaccine candidate strain with a truncated NS1 gene was constructed, verified by animal experiments that provide good protection for both specific pathogen-free (SPF) chickens (Chen et al., 2017). On the basis of this vaccine virus (rTX-NS1-128 strain), the HA packaging signal, including NCR, the 80 nucleotides (nt) of 5′ORF, and the 9 nt of 3′ORF (Watanabe et al., 2003), and the NS packaging signal, including NCR, 150 nt of 5′ORF, and 150 nt of 3′ORF (Fujii et al., 2005), were exchanged to avoid viral recombination and to construct safe and high-efficiency live attenuated vaccines.

## Materials and Methods

### Biosafety and Animal Care

4-week-old SPF chickens were purchased from Shandong Poultry Research Institute, China. All animal experiments *in vivo* were performed in the negative-pressure isolators of the authorized animal biosafety level 2 (ABSL-2) facilities at Yangzhou University, approved by the Jiangsu Administrative Committee for Laboratory Animals, and complied with the guidelines of laboratory animal welfare and ethics of the Jiangsu Administrative Committee for Laboratory Animals (Permission number: SYXKSU-2016-0020).

### Viruses and Cells

H9N2 AIVs A/chicken/Shanghai/F/98 (H9N2), A/chicken/Taixing/10/2010 (H9N2, rTX), and the NS1 truncated virus (rTX-NS1-128) (Chen et al., 2017) were identified and stored by our laboratory and were cultured in the 9- to 11-day-old embryonated SPF chicken eggs at 37°C. Allantoic fluids were harvested 72 h post-inoculation and stored at −70°C. Human embryonic kidney (293T) cells and Madin–Darby canine kidney (MDCK) cells were cultured in high glucose Dulbecco’s modified Eagles’ medium (DMEM) (HyClone, UT, United States) with 10% fetal bovine serum (FBS) (HyClone). Chicken embryo fibroblasts (CEF) cell were grown in M199 medium (HyClone, United States) with 4% FBS. The cells were incubated at 37°C with 5% CO_2_.

### Plasmid Construction

**FIGURE 1:**
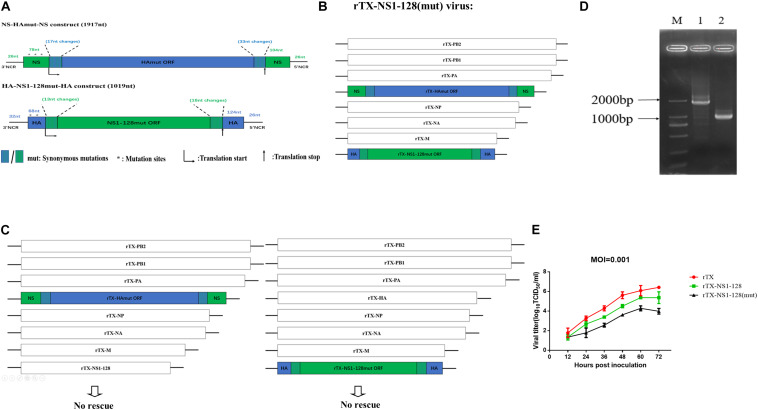
Construction of recombinant virus with the replacement of the packaging signal fragments and their growth kinetics. **(A)** Construction of NS-HAmut-NS and HA-NS1-128mut-HA. The 3′ and 5′ ends of rTX-NS1-128 HA ORF were amplified by synonymous mutant primers to generate 17-nt and 33-nt synonymous mutations, respectively (in blue). Then, the HA ORF was flanked by NS 3′ and 5′ NCRs and the 76 and 104 nt of NS ORF packaging signal (in green). Similarly, a 1,019-nt HA-NS1-128mut-HA was constructed. The ATGs (in a positive sense) upstream of the HA and NS translation start codons were all mutated to TTGs (in a positive sense). The 5’ splice site on the 57 nt of NS packaging signal in the NS-HAwt-NS was also mutated (see *Materials and Methods*). **(B)** Genome structure of the swap wild-type virus. PB2, PB1, PA, NP, NA, and M plasmids of TX strain, NS-HAmut-NS, and HA-NS1-128mut-HA plasmids were used to generate the rTX-NS1-128 (mut) virus. **(C)** The recombinant virus cannot be rescued independently using NS-HAmut-NS or HA-NS1-128mut-HA plasmid plus the other seven plasmids from TX. **(D)** The amplification of HA and NS genes of the rTX-NS1-128. NS-HAmut-NS was amplified by using primers to target the NCR of NS (lane 1), and HA-NS1-128mut-HA was amplified by using primers to target the NCR of HA (lane 2); lane M was the marker. **(E)** Viral growth kinetics in MDCK cells. MDCK cells were infected by rTX, rTX-NS1-128, and rTX-NS1-128 (mut) with MOI 0.001. The supernatant was collected every 12 h, and TCID_50_/ml was titrated on CEF cells. The mean and standard errors are shown from three independent experiments.

pHW291-PB2, pHW292-PB1, pHW293-PA, pHW294-HA, pHW295-NP, pHW296-NA, pHW297-M, pHW298-NS (GenBank Taxonomy ID: 1082519), and pHW298-NS1-128 were previously constructed by Hoffmann’s method (Hoffmann et al., 2001; Chen et al., 2017). The ORF of the HA gene with synonymous mutations was amplified from the pHW294-HA plasmid and then transferred into the Blunt3 vector (TransGen Biotech, China). The pHW298-NS1-128 plasmid was subjected to site-directed mutagenesis to mutate four ATGs (A27T, A76T, A727T, and A795T) and one splice site (G57C). The NS1 packaging signal was amplified from the site-directed mutation pHW298-NS1-128. The ORF-mut of the TX HA [1,683 base pairs (bp)], pHW2000 (2,961 bp), 3′ NS packaging signal (104 bp), and 5′ NS packing signal (130 bp) were amplified and ligated by one step cloning kit (Beijing Biodragon, China), and the recombinant plasmid was named NS-HAmut-NS. Recombinant plasmid HA-NS1-128mut-HA was generated by the method that is similar to NS-HAmut-NS. Briefly, the ORF mutation of TX NS1-128 with synonymous mutation amplified from pHW298-NS1-128 and the HA packaging signal with site-directed mutagenesis (five ATGs: A33T, A82T, A1647T, A1677T, and A1697T) were ligated to pHW2000 together (Figure 1A and Table 1).

**TABLE 1:** Primers for the construction of mutant plasmids of NS-HAmut-NS and HA-NS1-128mut-HA.

Primer name	Primer sequences (5′→3′)	Description
HA ORF-F	atg gaA acC atC tcC ctC atC acC atC ctG ctG gtC gtC acG gtC TCG aat gca gac aaa atc tgc at	The ORF of HA and NS1 with synonymous mutations
HA ORF-R1	cca Aaa TaG Aaa Agc Ggc Gaa Tcc cat Ggc Gat Tac Gag gCT GCT Tgc gac agt cga ata aat ggt g	
HA ORF-R2	tca Gat Gca Gat Att Aca Cct Aca GCT Gcc Gtt gCT cat Cgc cca Aaa Tag Aaa Agc Ggc Gaa Tc	
NS1-128 ORF-F	atg gaC AGC aaT acC gtC AGT TCT ttc cag gta gaT tgc ttt ctt tgg cat gtc c	
NS1-128 ORF-R	tta Gat CaA Ttg Gaa GCT Aaa Ggt CcG Gat Ttc Ctg Ctc cac ttc aag c	
HA A33T F	ctc aag Ttg gag aca ata tca cta ata act ata cta cta gta g	The packaging signal of HA and NS1 with synonymous mutations
HA A33T R	gat att gtc tcc aAc ttg agt gtt gtg aaa ttc ccc tgc ttt	
HA A82T F	aca gta agc aTt gca gac aaa atc tgc atc ggc t	
HA A82T R	ttt gtc tgc aAt gct tac tgt tac tac tag tag tat ag	
HA A1647T F	tgt gat tgc aTt ggg gtt tgc tgc ctt ctt gtt	
HA A1647T R	gca aac ccc aAt gca atc aca agg gat gag gcg	
HA A1677T F	gtt ctg cgc ctt gtc caT tgg gtc ttg c	
HA A1677T R	acc caA tgg aca agg ccc aga aca aga ag	
HA A1697T F	ggt ctt gca gTt gca aca ttt gta ttt gat tgg caa aaa c	
HA A1697T R	caa atg ttg caA ctg caa gac cca atg gac aag g	
NS1-128 A27T F	cat aTt gga ttc caa cac tgt gtc aag ctt c	
NS1-128 A27T R	cag tgt tgg aat cca Ata tgt ttt tgt cac cct gct ttt gct	
NS1-128 G57C F	aag ctt cca gCt aga ctg ctt tct ttg gct tgt ccg	
NS1-128 G57C R	aag cag tct aGc tgg aag ctt gac aca gtg ttg g	
NS1-128 A76T F	ttt ctt tgg cTt gtc cgc aaa cga ttt gca t	
NS1-128 A76T R	ttt gcg gac aAg cca aag aaa gca gtc tac ctg g	
NS1-128 A727T F	aat aac ttt tTt gca agc ctt aca act att gct tga ag	
NS1-128 A727T R	gct tgc aAa aaa gtt att tgc tca aag cta ttt tcc gta att t	
NS1-128 A795T F	agc tta ttt tTt gat aaa aaa cac cct tgt ttc tac t	
NS1-128 A795T R	ttt ttt atc aAt aaa taa gct gaa acg aga aag ttc	

*Uppercase indicates the synonymous mutation.*

### Reverse Genetics for Recombinant Viruses

To generate the rTX-NS1-128 (mut) virus, co-cultured 293T/MDCK (3:1) cells were transfected with six TX backbone plasmids (pHW291-PB2, 292-PB1, 293-PA, 295-NP, 296-NA, and 297-M) and chimeric HA and NS constructs (NS-HAmut-NS and HA-NS1-128mut-HA), based on a previous report by Hoffmann et al. (2000). Three hundred nanograms of plasmids encoding each gene segment was added into 50 μl of DMEM and incubated with 3 μl of PolyJet DNA Transfection Reagent (SignaGen Laboratories, United States) at room temperature for 20 min. The transfection complexes were transferred to co-cultured 293T/MDCK (1:3) to 80% in 35-mm dishes and incubated at 37°C with 5% CO_2_ for 8 h. Transfection supernatants were replaced with 1.5 ml of DMEM supplemented with 2 μg/ml TPCK-trypsin (Worthington, Lakewood, NJ, United States). At 72 h post-transfection, the cell supernatants were collected and freeze-thawed three times to inoculate into 9-day-old SPF embryonated chicken eggs to amplify the viruses. Allantoic fluid samples were collected and continued to passage to the 15th generation. The HA and NS sequences of the 15th passage viruses were confirmed by sequencing.

### Viral Growth Kinetics

MDCK cells (1 × 10^6^/well) were cultured in six-well plates and infected with rTX, rTX-NS1-128, or rTX-NS1-128 (mut) virus at a multiplicity of infection (MOI) of 0.001. One-hour post-infection (hpi), the inoculum was removed, and the plates were washed twice with PBS and replaced with 2 ml of DMEM supplemented with 2 μg/ml TPCK-trypsin. Supernatants were collected at 12, 24, 36, 48, 60, and 72 hpi and titrated the median tissue culture infectious dose (TCID_50_) of each supernatant on chicken embryo fibroblast (CEF) cells.

10-day-old SPF embryonated chicken eggs were inoculated with 10^–5^–10^–10^ diluted virus, incubated at 37°C for 72 h, the hemagglutination (HA) titer of allantoic fluid was titrated, and the median egg infectious dose (EID_50_) was calculated by Reed and Muench method (Reed and Muench, 1938).

### Reassortment Efficiency Detection

MDCK cells (1 × 10^6^/well) were cultured in 6-well plates and co-infected with the rTX-NS1-128 (mut) virus and rTX virus at an MOI of 10. At 1 hpi, the plates were washed three times with PBS and replaced with 1.5 ml of DMEM supplemented with 2 μg/ml TPCK-trypsin. Supernatants were collected to perform the plaque assay after 12 hpi.

The supernatants were freeze-thawed three times and diluted to 10^–5^ with Minimum Eagle’s Medium (MEM) (HyClone, United States). After 200 μl dilution was added to single-layer MDCK cells, the 12-well plate was incubated for 1 h at 37°C and was shaken and mixed every 15 min during this process. The inoculum was replaced with 1 ml/well of 2 × EMEM (Genom, China) with 2 μg/ml TPCK-trypsin (Worthington-Biochem, United States) plus 1.6% agarose solution, which was solidified at room temperature and then incubated at 37°C. After 60 h, every well added 1 ml 1:20 diluted mixture solution (0.33% neutral red solution and 1.6% agarose water with 1:1). After 24 h, single plaques were isolated from amplification in MDCK cells. At 60 hpi, RNA was purified from the amplified virus, then the HA and NS were detected by reverse transcription-polymerase chain reaction (RT-PCR).

### Viral Pathogenicity in Chickens

The virulence of the recombinant viruses was determined in 4-week-old SPF chickens. Chickens (*n* = 16/group) were intranasally inoculated with 10^6^ EID_50_ of rTX-NS1-128 or rTX-NS1-128 (mut) in 0.2 ml, respectively. Three inoculated chickens per group were sacrificed on 3 and 5 dpi, and tracheas and lungs were collected and fully ground according to the standard of 1 g tissue sample/0.3 ml PBS for virus isolation and EID_50_ detection. Besides, oropharyngeal and cloacal swabs of 10 inoculated chickens were also collected, and the sample extracts were inoculated into embryonated SPF embryonated chicken eggs for virus titration on 3 and 5 dpi.

### The Viral Transmissibility in Chickens

To determine the viral transmission, two groups of 4-week-old SPF chickens (*n* = 10/group) were intranasally inoculated with 10^6^ EID_50_ viruses of rTX-NS1-128 or rTX- NS1-128 (mut) in 0.2 ml. Then, the five naive in-contact chickens were placed in each group to contact the inoculated chickens for 24 h. Oropharyngeal and cloacal swabs were collected, and the sample extracts were inoculated into embryonated SPF embryonated chicken eggs for virus titration on 3 and 5 days post-inoculation or contact. Serum samples were collected to determine the antibody titer by hemagglutination-inhibition (HI) assay on 14 days post-inoculation or contact.

### Cytokine Expression and IgA and IgG Secretion in the Respiratory Mucosa

To determine the recombinant viruses’ ability to induce cellular immunity and mucosal immunity, two groups of 3-week-old SPF chickens (*n* = 25/group) were inoculated intranasally with 0.2 ml of 10^6^ EID_50_ rTX-NS1-128 (mut) virus or subcutaneously with 0.2 ml of oil-emulsified inactivated virus rTX. Five chickens were sacrificed on 1 and 3 dpi, and the nasal and trachea samples were collected. After homogenization with PBS, the supernatant samples were collected to extract RNA. Real-time quantitative reverse transcription PCR (qRT-PCR) was used to detect relative expression levels of IL-2, IL-6, and IL-12 (Table 2). On 7, 14, and 21 dpi, five chickens were sacrificed, and nasal and trachea samples were repeatedly washed with 0.5 ml of PBS. The supernatant samples were collected after centrifugation of the lavage fluid to detect relative expression levels of chicken specific secretory IgA and IgG by ELISA kit (Laierbio, China).

**TABLE 2:** Primers of quantitative real-time PCR for the detection of mRNA level of the main cytokines of chickens.

Target gene	Forward primer	Reverse primer
IL-2	GAACCTCAAGAGTCTTACGGGTCTA	ACAAAGTTGGTCAGTTCATGGAGA
IL-6	CGGCAGATGGTGATAAATCC	CCCTCACGGTCTTCTCCATA
IL-12	ACCAGCCGACTGAGATGTTC	GTGCTCCAGGTCTTGGGATA
β-actin	ATGAAGCCCAGAGCAAAAGA	GGGGTGTTGAAGGTCTCAAA

### Vaccination and Challenge in Chickens

Two groups of 4-week-old SPF chickens (*n* = 10/group) were vaccinated intranasally with 0.2 ml of 10^6^ EID_50_ rTX-NS1-128 (mut) virus, and the control group was inoculated with PBS. Serum was collected every week after inoculation for antibody titration and was detected until 11 weeks after immunization.

Three weeks after injection, vaccinated chickens were challenged intranasally with 10^6^ EID_50_ of TX (homologous) or F98 (heterogeneous) H9N2 subtype AIV. On 3, 5, and 7 dpi, chickens were monitored daily for morbidity and mortality after challenge, and the oropharyngeal and cloacal swabs were collected to determine viral titer by SPF embryonated chicken eggs.

### Statistical Analysis

Comparisons of experimental groups were estimated using *t* tests with a two-tailed analysis to determine significant differences. Pearson’s chi-square test or Fisher’s exact test was applied to compare the positive swab rate. The software GraphPad Prism version 6 (GraphPad Software, San Diego, CA, United States) was used to make the graphs. The data are shown as the mean fold change ± SD of the results. Statistical significance was denoted by the symbol ^∗^(*P* < 0.05).

## Results

### The rTX-NS1-128 (mut) Recombinant Virus Was Successfully Constructed and Rescued

We constructed NS-HAmut-NS and HA-NS1-128mut-HA plasmids, and the lengths were 1,917 and 1,019 bp, respectively. By using the transfection system, we rescued the rTX-NS1-128 (mut) virus, which contained six A/chicken/Taixing/10/2010 (H9N2) segments (PB2, PB1, PA, NP, NA, and M) and two chimeric segments NS-HAmut-NS and HA-NS1-128mut-HA (Figure 1B), named rTX-NS1-128 (mut). To determine whether the chimeric segments can independently reassort with wild-type segments, we attempted to rescue two viruses containing seven TX segments and single chimeric segments, however, these two viruses were unable to be rescued (Figure 1C). The rescue’s failure indicated that the HA-NS1-128mut-HA or NS-HAmut-NS segments could not freely reassort with wild-type virus segments.

The rescued recombinant virus was confirmed by RT-PCR and sequencing of HA and NS genes (Figure 1D). The rTX-NS1-128(mut) was passaged at least 15 generations in 10-day-old SPF embryonated chicken eggs, and then the sequencing results showed no difference between the 15th generation and the first rescued recombinant virus.

### The rTX-NS1-128 (mut) Recombinant Virus Possessed Good Growth Kinetics

Growth properties of the rTX-NS1-128(mut), rTX-NS1-128, and rTX were compared in MDCK cells and 10-day-old SPF embryonated chicken eggs. All viruses replicated well in eggs with their titers 8.41 ± 0.29, 8.16 ± 0.29, and 7.83 ± 0.35 log_10_EID_50_/0.1 ml, respectively; the titer of all mutant viruses was slightly lower than that of wild-type virus (rTX), while there was no significant difference among them (*P* > 0.05) (Table 3). The growth properties of two recombinant viruses in MDCK cells were lower than those of rTX. rTX-NS1-128 (mut) titers peaked at 60 hpi (between 10^4^ and 10^5^ TCID_50_/ml) and were significantly lower than that of wild-type virus (10^6^ TCID_50_/ml) (Figure 1E).

**TABLE 3:** EID_50_ of rescued viruses.

Virus	Log_10_EID_50_/0.1 ml
rTX	8.41 ± 0.29
rTX-NS1-128	8.16 ± 0.29
rTX-NS1-128 (mut)	7.83 ± 0.35

### Replacement of the Packaging Signals Effectively Prevented the Viral Reassortment

**FIGURE 2:**
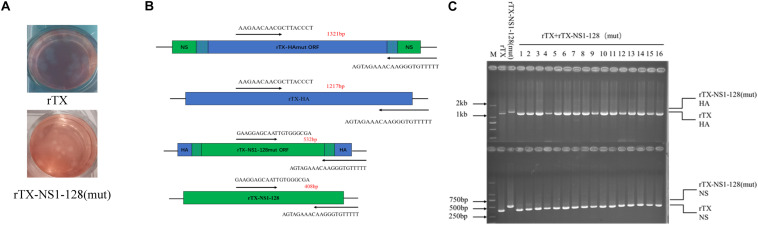
Reassortment efficiency of rTX-NS1-128 (mut) virus. **(A)** Co-infection supernatants of rTX and rTX-NS1-128 (mut) in MDCK cells were collected for plaque analysis and plaque morphology, as shown. **(B)** Primers were designed to detect chimeric segments or original segments, and the RT-PCR products are 1,321 and 1,217 bp for HA, and 532 and 408 bp for NS, respectively. **(C)** The rTX-NS1-128 (mut) and rTX co-infection experiment. Forty-eight single plaques were characterized by RT-PCR (16 representative plaques were shown). All of the viruses evaluated were rTX.

The single plaque (Figure 2A) was picked up, and HA and NS segments were detected by RT-PCR. A 1,321 bp product was observed for NS-HAmut-NS segments, while for the rTX, a 1,217 bp band can be obtained. The PCR products for chimeric and wild-type NS segments, on the other hand, were 532 and 408 bp, respectively (Figure 2B). After performing the co-infection of rTX-NS1-128 (mut) and rTX, 48 plaques were picked. We found that all of the plaques contained both wild-type HA and NS genes, indicating the inability of NS-HAmut-NS or HA-NSmut-HA to reassort freely, which illustrates the selection of wild-type virus rTX over reassortant virus rTX-NS1-128 (Figure 2C).

### The rTX-NS1-128 (mut) Recombinant Virus Has Low Pathogenicity in Chickens

To examine mutant viral pathogenicity, chickens were inoculated intranasally with 10^6^ EID_50_ of the rTX-NS1-128 (mut) and rTX-NS1-128 viruses. Until 14 dpi, no infected chickens were found to have clinical signs of H9N2 AI.

In trachea tissues on 3 and 5 dpi, the rTX-NS1-128 virus was detected in two of three chickens, the rTX-NS1-128 (mut) virus was detected in one of three chickens, and there was no significant difference between rTX-NS1-128 (mut) and rTX-NS1-128 (*P* > 0.05). In the lungs, no viruses were detected (Table 4).

**TABLE 4:** Viral titers in trachea and lung tissues of SPF chickens.

Virus	Trachea (log_10_EID_50_/0.3 g)		Lung (log_10_EID_50_/0.3 g)	
	3 dpi	5 dpi	3 dpi	5 dpi
rTX-NS1-128	(2/3)^a^ 2.0 ± 0.28	(2/3) 0.81 ± 0.2	ND^b^	ND
rTX-NS1-128 (mut)	(1/3) 2.77	(1/3) 2.33	ND	ND

*^a^No. of positive chickens/total chickens used. ^b^Not detected.*

Viral shedding levels of rTX-NS1-128 and rTX-NS1-128(mut) were detected by the titration of oropharyngeal and cloacal swabs. On 3 dpi, rTX-NS1-128 and rTX-NS1-128 (mut) viruses were all shedding from the oropharynx, and the viral shedding rate was 80 and 100%, respectively. On 5 dpi, viruses were still shedding from the oropharynx, and the shedding rate of the two viruses was the same: both were 80%. On 7 dpi, the shedding rates of rTX-NS1-128 and rTX-NS1-128(mut) were reduced to 30 and 20%, respectively (Table 5).

**TABLE 5:** Virus shedding of SPF chickens inoculated with recombinant viruses.

Virus	3 dpi		5 dpi		7 dpi	
	O^a^	C^b^	O	C	O	C
rTX-NS1-128	10/10 ^c^	0/10	8/10	0/10	3/10	0/10
rTX-NS1-128 (mut)	8/10	0/10	8/10	0/10	2/10	0/10

*^a^Oropharyngeal swab. ^b^Cloacal swab. ^c^No. of positive chickens/total chickens used.*

### *C*ontact *T*ransmission of rTX-NS1-128 (mut) *R*ecombinant *V*irus Did Not *O*ccur in *C*hickens

Two groups of chickens were inoculated intranasally with rTX-NS1-128 (mut) and rTX-NS1-128 with 10^6^ EID_50_. On 1 dpi, five chickens were housed with virus-inoculated chickens as contact groups. On 3 and 5 dpi, the rTX-NS1-128 and rTX-NS1-128(mut) can be detected in the oropharynx of directly inoculated groups. There was no significant difference between rTX-NS1-128(mut) and rTX-NS1-128 (*P* > 0.05). Besides, rTX-NS1-128 (mut) and rTX-NS1-128 were not detected from oropharynx samples in contact chickens. Meanwhile, viruses led to seroconversion in all inoculated chickens but not contact chickens on 14 dpi (Table 6).

**TABLE 6:** Transmission characteristics of recombinant viruses in SPF chickens.

Virus	Dose EID_50/0.1ml_	Method of transmission	3 dpi		5 dpi		Sero-conversion^c^
			O^a^	C^b^	O	C	
rTX-NS1-128	10^6^	Inoculation	8/10 ^d^	0/10	5/10	0/10	+(10/10)
		Contact	0/5	0/5	0/5	0/5	−(0/5)
rTX-NS1-128(mut)	10^6^	Inoculation	6/10	0/10	4/10	0/10	+(10/10)
		Contact	0/5	0/5	0/5	0/5	−(0/5)

*^a^Oropharyngeal swab. ^b^Cloacal swab. ^c^HI titer of chickens 14 days post-inoculation or post-exposure. HI < 4log2 was considered negative. ^d^No. of positive chickens/total chickens used.*

### rTX-NS1-128 (mut) Recombinant Virus Can Strongly Induce Cellular and Mucosal Immunity

**FIGURE 3:**
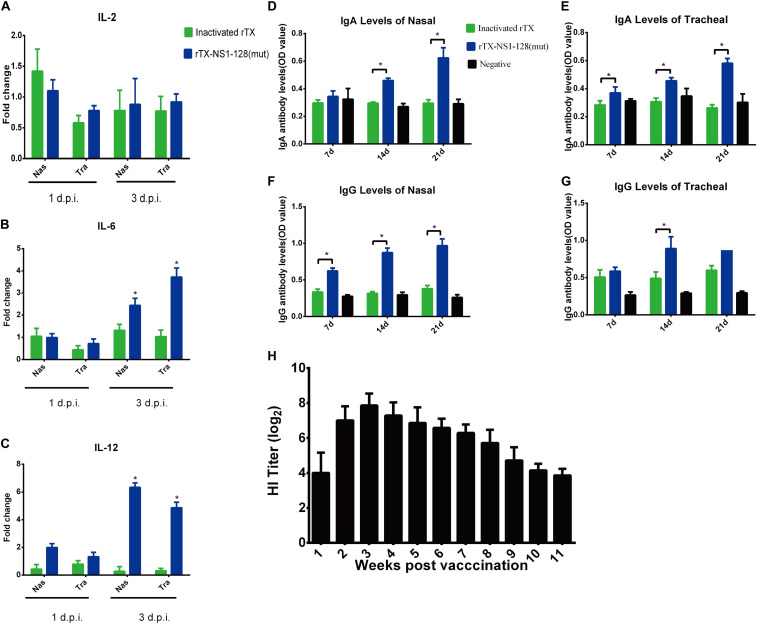
Detection of cytokine genes, IgA, IgG, and HI antibody levels. 3-week-old SPF chickens (*n* = 25/group) were prepared and inoculated intranasally with 10^6^ EID_50_ rTX-NS1-128 (mut) virus or subcutaneously oil-emulsified inactivated virus rTX in 0.2 ml, respectively; negative controls were set. **(A–C)** Five chickens were euthanized, and the nasal and trachea samples were collected on 1 and 3 dpi. The relative expression levels of IL-2, IL-6, and IL 12 were detected by qRT-PCR. **(D–G)** Five chickens were euthanized on 7, 14, and 21 dpi; nasal and trachea samples were repeatedly washed with 0.5 ml of PBS. The supernatant was collected to detect the levels of chicken specific secretory IgA and IgG by using an ELISA kit. Data represent the mean fold changes ± standard errors. *P < 0.05; **P < 0.01, compared to the value of the rTX. **(H)** Ten chickens were inoculated with 10^6^ EID_50_ rTX-NS1-128 (mut) virus in 0.2 ml, and the serum was separated at 1-week intervals after inoculating. HI titers in sera were determined.

On 1 and 3 dpi, the nasal and tracheal tissues were collected, and the relative expression of cytokines was detected by qRT-PCR. The results of IL-2 levels in both tracheal and nasal tissues showed that there was no significant difference between rTX and rTX-NS1-128 (mut) (*P* > 0.05, Figure 3A). The IL-6 expression levels of the rTX-NS1-128 (mut) live virus group were significantly higher than that of the rTX inactivated virus group on 3 dpi (*P* < 0.05, Figure 3B). On 1 dpi, the IL-12 expression levels of the rTX-NS1-128 (mut) group increased compared with that of the rTX inactivated virus group, and on 3 dpi, it was significantly higher than that of rTX inactivated virus group (*P* < 0.05) (Figure 3C). ELISA results showed that the tracheal IgA and IgG levels from the group of rTX-NS1-128 (mut) were significantly higher than the inactivated virus group on 14 and 21 dpi (*P* < 0.05, Figures 3D,E). Similarly, the nasal IgA and IgG levels of rTX-NS1-128 (mut) show a significant increase compared with those of the inactivated virus group on 14 and 21 dpi (*P* < 0.05, Figures 3F,G).

### rTX-NS1-128 (mut) Recombinant Virus Can Provide Good Protection Against Both Homologous and Heterologous Virus

4-week-old SPF chickens were inoculated intranasally with 10^6^ EID_50_ of rTX-NS1-128(mut). Hemagglutination inhibition (HI) titers increased rapidly to 7 log2 at 2 weeks post-inoculation (wpi) and peaked to 7.86 log2 at 3 wpi. High HI titers lasted for about 8 weeks and then sharply decreased to 3.86 log2 at 11 wpi (Figure 3H). Moreover, chickens were challenged with homologous (TX strain) and heterologous (F98 strain) H9N2 virus on 21 dpi. Virus shedding was detected in the two oropharynx samples from the rTX-NS1-128(mut)-immunized chickens after challenging F98 on 3 dpi. No virus was shed from rTX-NS1-128(mut)-immunized chickens after challenging with TX on 3, 5, and 7 dpi. However, the viruses were detected in the oropharynx samples from all control groups 3 days after challenge (Table 7). These data suggested that rTX-NS1-128 (mut) can provide protections against both homologous and heterologous H9N2 virus attack in chickens.

**TABLE 7:** Protective efficacy of candidate vaccines in SPF chickens.

Group	Quantity	Challenge virus	3 dpi		5 dpi		7 dpi	
			O^a^	C^b^	O	C	O	C
rTX-NS1-128(mut)	10	F98(H9N2)	2/10^c^	0/10	0/10	0/10	0/10	0/10
rTX-NS1-128(mut)	10	TX(H9N2)	0/10	0/10	0/10	0/10	0/10	0/10
Control	10	F98(H9N2)	10/10	3/10	5/10	2/10	0/10	0/10
Control	10	TX(H9N2)	10/10	4/10	8/10	1/10	1/10	0/10

*^*a*^Oropharyngeal swab. ^*b*^Cloacal swab. ^*c*^No. of positive chickens/total chickens used.*

## Discussion

By exchanging the NS and HA packaging signals and removing the original packaging sequence in the ORF through synonymous mutations, the generating recombinant virus containing chimeric HA and NS fragments will not be able to reassort freely with NS and HA fragments of wild-type virus (Gao and Palese, 2009). We have previously constructed a strain of NS1 truncated H9N2 virus, named rTX-NS1-128, which exhibited an attenuated phenotype, lost transmissibility, good immunogenicity, and reactogenicity (Chen et al., 2017). In this study, we exchanged the packaging sequences of HA and NS1-128 segments based on the rTX-NS1-128 strain, eliminated original packaging sequences of ORF, and successfully rescued the recombination virus rTX-NS1-128 (mut).

Two recombinant plasmids (NS-HAmut-NS and HA-NS1-128mut-HA) were replaced into the TX backbone to obtain a recombinant virus rTX-NS1-128 (mut). Replacing either of two recombinant plasmids will cause a viral rescue failure, consistent with a recombined PR8 strain (Gao and Palese, 2009). These data implied that the recombinant HA and NS genes could not independently reassort HA and NS genes of the wild-type virus. The packaging signals of the HA and NS1 truncated genes were only replaced in the recombinant plasmids; therefore, the recombinant genes’ assembly into the parent virus will not affect the interaction with the other six segments of the packaging signal vRNAs, and the recombinant virus can be successfully rescued. However, when trying to replace the H5 backbone, even if two plasmids were replaced, the virus cannot be rescued. A dominant hypothesis regarding virus assembly is through the interaction of different fragments of vRNAs (Fujii et al., 2003). Recent studies have also shown that the packaging of the IAV genome is a selective process, eight vRNA fragments interact to form a macromolecular complex (Haralampiev et al., 2020). In other words, it is difficult for each packaging signal to play an independent role.

The incompatibility between RNA packaging signals is sufficient to prevent the formation of specific reassortant genotypes (Essere et al., 2013). Two artificial vRNAs with the same packaging signal but encoding different proteins compete with each other to be packaged into virions, thereby packaging a single copy of vRNA (Inagaki et al., 2012). The HA and NS1 that exchange packaging signals need to compete with HA and NS1 that have natural packaging signals. The results of recombination efficiency showed that the HA and NS1 of rTX-NS1-128 (mut) have no competitive advantage compared with wild-type strains (Figure 2C), which is consistent with the recombined PR8 strain (Gao and Palese, 2009). Some studies have shown that the probability of single-segment recombination is around 20% in a co-infection experiment of influenza viruses *in vitro* (Marshall et al., 2013; Zhou et al., 2017). In addition to HA and NS1, the H9N2 virus can provide internal genes to new emerging reassortant viruses; for instance, the novel reassortant H7N9 virus emerged in China in 2013 (Wu et al., 2013). Therefore, a similar approach could be used to create a virus that completely lack recombination capabilities. For example, PB2 has an HA packaging signal, PB1 has an NA packaging signal, PA has an PB2 packaging signal, etc.

The previous study showed that the packaging signals only guaranteed a maximum packaging efficiency with 60%, indicating that the packaging signal sequence is not limited to the 5′ and 3′ regions (Watanabe et al., 2003; Fujii et al., 2005), which may be why the titer of rTX-NS1-128 decreased on MDCK cells and chicken embryos compared with the parent virus.

The pathogenicity of the recombinant virus and the parental virus were compared, and the results were consistent with the data of rTX-NS1-128 (Chen et al., 2017), indicating that the recombinant virus still had an attenuated phenotype after the exchange of packaging signals. H9 subtype AIV can be transmitted through aerosols in chickens (Yao et al., 2014). Previous studies have confirmed that the parental virus TX can be transmitted by contact, while the virus rTX-NS1-128 with truncated NS gene can block the virus to contact transmission (Chen et al., 2017). Consistently, our results indicated that the recombinant virus rTX-NS1-128 (mut) was still not able to spread through contact after the exchange of packaging signals.

Most of the live attenuated viruses can induce similar humoral immunity levels compared to inactivated viruses; however, it can effectively induce a stronger mucosal immunity and a broader and faster cellular immune response (Hoft et al., 2011). Intranasal immunization is a great potential strategy based on its needle-free delivery, as well as production of mucosal antibodies at the site of entry for the influenza virus to neutralize and prevent transmission (Qin et al., 2020). Compared with inactivated rTX, rTX-NS1-128 (mut) can remarkably increase the level of IL-6, suggesting that Th-2 immune response can be effectively stimulated. Cellular immunity around the mucosa also plays an important role at the early stage of infection. A previous study indicated that live viruses contribute to promoting the activation of cytokines, such as IL-2 and IL-12, which activate cells to secrete IFN-γ or stimulate T cell response (Rauw et al., 2010). Our results showed that rTX-NS1-128 (mut) can induce higher mucosal and cellular immune compared to inactivated rTX, suggesting that the exchange of packaging signals does not affect the virus’ ability to induce immunity. A large number of previous studies have shown that the inactivated H9N2 vaccine cannot provide strong cellular immunity and mucosal immunity, resulting in a low protection rate for heterologous strains (Sun et al., 2012; Shin et al., 2016; Wei et al., 2016b). Our recombinant virus with exchanged packaging signals protects against the homologous H9N2 virus and a heterologous H9N2 virus demonstrating broad influenza virus immunity.

In summary, rTX-NS1-128 (mut) can induce good immune protection and has the characteristics of low pathogenicity, non-transmission, good genetic stability, and prevention of recombination. rTX-NS1-128 (mut) could potentially be a safer live attenuated vaccine candidate.

## Data Availability Statement

The raw data supporting the conclusions of this article will be made available by the authors, without undue reservation.

## Ethics Statement

The animal study was reviewed and approved by Jiangsu Administrative Committee for Laboratory Animals.

## Author Contributions

SC and DP conceived and designed the experiments. SC, HW, SL, and JX performed the experiments. SC, KQ, and TQ analyzed the data. SC, KQ, HW, TQ, DC, GF, and YD contributed reagents, materials, and analysis tools. SC and KQ wrote the manuscript. SC, KQ, DP, and TQ designed the program used in the analysis. All authors contributed to the article and approved the submitted version.

## Conflict of Interest

DC, GF, and YD were employed by the Qingdao Yibang Biological Engineering Co., Ltd.,(Qingdao, China). The remaining authors declare that the research was conducted in the absence of any commercial or financial relationships that could be construed as a potential conflict of interest.
